# Long-term efficacy and safety of atazanavir/ritonavir treatment in a real-life cohort of treatment-experienced HIV patients

**DOI:** 10.1186/1758-2652-13-S4-P31

**Published:** 2010-11-08

**Authors:** K Jansen, A Sönnerborg, P Pugliese, S Biguenet, JL Eychenne, NH Brockmeyer, C Michalik, S Dupke, H Jaeger, A Plettenberg, D Butcher, MJ Jiménez-Expósito, HIV KompNet

**Affiliations:** 1 Competence Network for HIV/AIDS, Bochum, Germany; 2 Karolinska Institutet, Karolinska University Hospital, Department of Infectious Diseases, Stockholm, Sweden; 3 Nice University Hospital, Infectious Diseases Department, Nice, France; 4 Bristol-Myers Squibb Company, Wallingford, CT, USA; 5 Hays Pharma, Paris, Franc; 6 Clinic for Dermatology, Venerology, and Allergolog, Competence Network for HIV/AIDS, Bochum, Germany; 7 Clinical Trial Centre, Cologne, Germany; 8 Clinical Centre Driesener Straße, Berlin, Germany; 9 MVZ Karlsplatz, Munich, Germany; 10 Ifi-Institut, Hamburg, Germany;; 11 Bristol-Myers Squibb Company, Paris, France

## Purpose of the study

Atazanavir (ATV)-based regimens have demonstrated efficacy and safety in both ARV-naïve and -experienced patients. However, few reports have assessed effectiveness beyond 2 years. The aim of this study was to describe the long-term outcomes of ATV/r containing regimens in ARV-experienced patients in a clinical setting.

## Methods

Non-comparative, retrospective, observational study which collected data from 3 European databases (France-DatAids, Germany-KompNet, Sweden-InfCare). Clinical data for ARV-experienced adult patients who started an ATV/r-based regimen between October, 2004 and March, 2007 were extracted every 6-months (maximum follow-up 5 years). Primary endpoint was the proportion of patients remaining on ATV treatment by baseline HIV-1 RNA (< 500 or ≥ 500 c/mL). Secondary endpoints included virologic response and reason for discontinuation. The duration of treatment and time to virologic failure were analyzed using the Kaplan-Meier method.

## Summary of results

**Fig 1:**
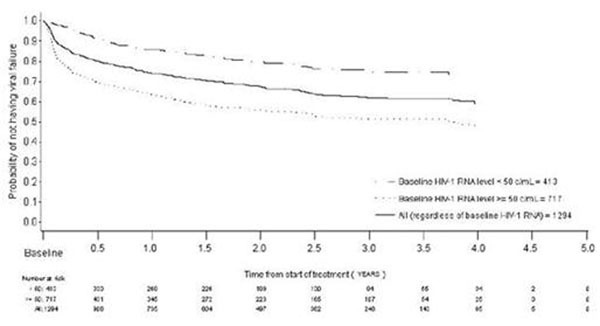
Time to virologic failure (two consecutive HIV-1 RNA ≥ 50 c/mL or one HIV-1 RNA ≥ 50 c/mL followed by discontinuation)

Data for 1294 ARV-experienced patients (prior ARV exposure: mean, 5.70 years) were analyzed. Patients were predominantly male (74%); median age 43 years (min, max: 18, 85); 75% had prior exposure to PIs (mean: 3.5 years). At baseline (BL), 56% of patients had HIV-1 RNA < 500 c/mL and 37% had < 50 c/mL. The estimated proportion of patients remaining on ATV during the follow-up period was 52% (median duration of treatment: 3.7 years); 54% for patients with BL HIV-1 RNA < 500 c/mL and 50% for those with BL HIV-1 RNA > 500 c/mL. The estimated probability of discontinuation was 21% during the first year and decreased at each subsequent 1-year treatment interval. Time to virologic failure is presented in Figure 1.

The most frequent reasons for discontinuation were "unknown" (32%), adverse events (25%), patient withdrew consent (13%) and lack of efficacy (11%). Hyperbilirubinemia was reported as reason for discontinuation in 12 patients. No unexpected changes in metabolic parameters were observed and renal AEs were reported rarely (1.9/100 patient-years)•

## Conclusions

In real life setting, ATV/r-based regimen demonstrated sustained virological efficacy in an ARV-experienced population including patients with previous virological failure. After long-term treatment a high proportion of patients remained on an ATV regimen and no unexpected AEs were observed.

